# The General Health Questionnaire-28 (GHQ-28) as an outcome measurement in a randomized controlled trial in a Norwegian stroke population

**DOI:** 10.1186/s40359-019-0293-0

**Published:** 2019-03-22

**Authors:** Ellen G. Hjelle, Line Kildal Bragstad, Manuela Zucknick, Marit Kirkevold, Bente Thommessen, Unni Sveen

**Affiliations:** 1 Department of Nursing Science, and Research Center for Habilitation and Rehabilitation Services and Models (CHARM), Faculty of Medicine, University of Oslo, Oslo, Norway; 2 Department of Geriatric Medicine, Oslo University Hospital, Oslo, Norway; 3 Oslo Centre for Biostatistics and Epidemiology, Department of Biostatistics, Faculty of Medicine, University of Oslo, Oslo, Norway; 4 Department of Neurology, Akershus University Hospital, Lorenskog, Norway; 5 Department of Geriatric Medicine and Physical Medicine and Rehabilitation, Oslo University Hospital, Oslo, Norway; 6 Faculty of Health Sciences, Oslo Metropolitan University, Oslo, Norway

**Keywords:** Factor analysis, Psychometric properties, Stroke, Quality of life

## Abstract

**Background:**

Several studies have documented the variety of post-stroke psychosocial challenges, which are complex, multifaceted, and affect a patient’s rehabilitation and recovery. Due to the consequences of these challenges, psychosocial well-being should be considered an important outcome of the stroke rehabilitation. Thus, a valid and reliable instrument that is appropriate for the stroke population is required. The factor structure of the Norwegian version of GHQ-28 has not previously been examined when applied to a stroke population.

The purpose of this study was to explore the psychometric properties of the GHQ-28 when applied in the stroke population included in the randomized controlled trial; “Psychosocial well-being following stroke”, by evaluating the internal consistency, exploring the factor structure, construct validity and measurement invariance.

**Methods:**

Data were obtained from 322 individuals with a stroke onset within the past month. The Kaiser-Meyer-Olkin (KMO) test was used to test the sampling adequacy for exploratory factor analysis, and the Bartlett’s test of sphericity was used to test equal variances. Internal consistency was analysed using Cronbach’s alpha. The factor structure of the GHQ-28 was evaluated by exploratory factor analysis (EFA), and a confirmatory factor analysis (CFA) was used to determine the goodness of fit to the original structure of the outcome measurement. Measurement invariance for two time points was evaluated by configural, metric and scalar invariance.

**Results:**

The results from the EFA supported the four-factor dimensionality, but some of the items were loaded on different factors compared to those of the original structure. The differences resulted in a reduced goodness of fit in the CFA. Measurement invariance at two time points was confirmed.

**Conclusions:**

The change in mean score from one to six months on the GHQ-28 and the factor composition are assumed to be affected by characteristics in the stroke population. The results, when applying the GHQ-28 in a stroke population, and sub-factor analysis based on the original factor structure should be interpreted with caution.

**Trial registration:**

ClinicalTrials.gov, NCT02338869, registered 10/04/2014.

## Background

Stroke may cause a number of psychosocial challenges that affect a patient’s rehabilitation and recovery [1, 2]. Several studies have documented the variety of post-stroke psychosocial challenges, which are complex and multifaceted and may have different trajectories [3, 4]. Due to the consequences of these challenges for stroke rehabilitation, psychosocial well-being should be considered an important outcome of rehabilitation.

One instrument that has been widely used for screening and assessing mental symptoms and psychosocial well-being is the General Health Questionnaire (GHQ). The purpose of the instrument is to discover features that distinguish psychiatric patients from individuals who consider themselves to be healthy, and the questionnaire particularly targets the grey area between psychological sickness and health [5]. Based on the original 60-item version, several versions of GHQ have been constructed. The GHQ-28 was developed by Goldberg and Hillier in 1979 and is based on an exploratory factor analysis (EFA) of the original GHQ-60 [6].

The GHQ-28 is currently being applied as the primary outcome measurement in a study evaluating the effect of a psychosocial intervention on well-being after stroke [7]. The present study was part of this multicentre, prospective, longitudinal, randomized controlled trial.

The GHQ-28 is a self-administered instrument and is considered appropriate for research purposes [5]. This scaled version was intended for studies in which the investigators seek more information than that provided by a single severity score. In the construction of the GHQ-28, items were selected to cover four main areas: somatic symptoms, anxiety and insomnia, social dysfunction and severe depression [6]. The GHQ-28 focuses on breaks in normal function that lead to an inability to carry out one’s normal healthy activities. The questionnaire is concerned with the manifestation of new phenomena of a distressing nature within the last few weeks [5].

The GHQ-28 was originally developed in English for Londoners. The questionnaire has been translated into several different languages, including a Norwegian translation by Tom Andersen [8]. The dimensions of psychological health have been suggested to be universal across cultures [6]. The stability of the factor structures has been evaluated [9, 10] across different cultures and samples [11–14]. The stability has mostly been confirmed across several different centres, except for that in the study of Prady et al. They did not confirm goodness of fit to the original structure or measure invariance across different cultures [12].

Two studies have assessed the validity of the GHQ-28 for screening for post-stroke depression, in relation to diagnosis by a standardized psychiatric interview [15, 16]. The researchers found that patients with depression scored significantly higher on the GHQ-28 than non-depressed stroke patients. The only study found, that evaluated measurement invariance of GHQ-28 in a stroke population is that of Munyonbwe et al. [17], who evaluated measurement invariance prior to merging two samples for analysis. In their conclusion, the researchers established a strong measurement invariance in two different stroke populations and confirmed the original four-factor structure. They did not assess the measurement invariance over time, but recommended that future research on measurement invariance also evaluate if the same construct is being measured across different time points within samples [17].

In Norway, psychometric properties of the GHQ-30 version have been examined when used in a population of older people living at home [18]. In this study, the original factor structure of the GHQ-30 was supported. Sveen et al. [19] tested the factor structure of the 20-item version in patients who had suffered a moderate stroke. The factor analysis in that study generated three factors: anxiety, coping, and satisfaction. The factor structure of the Norwegian version of GHQ-28 has not previously been examined when applied to a stroke population.

Finding the right outcome measurement is an important aim when evaluating a complex intervention [20]. Culture and treatment vary between populations and countries. We believe that an investigation of the GHQ-28 when applied in a Norwegian stroke population are a valuable contribution to the knowledge of suitable outcome measurements for evaluating effect of psychosocial interventions in various stroke populations.

The aim of the present study was to explore the psychometric properties of the GHQ-28 when applied in a Norwegian stroke population by evaluating the internal consistency, exploring the factor structure, construct validity and measurement invariance.

## Methods

### Setting and study population

In total, 353 patients from 11 Norwegian acute stroke or rehabilitation units providing acute stroke care were included in the study from November 2014 to November 2016. The inclusion criteria were as follows: the participants should be 18 years of age or older, have suffered an acute stroke within the last month, be medically stable, be evaluated by the recruiting personnel to have sufficient cognitive functioning to participate, be able to understand and speak Norwegian, and be capable of giving informed consent. Exclusion criteria were having moderate to severe dementia, other serious somatic or psychiatric diseases, or severe aphasia.

### Data collection procedures

Data were collected at baseline (T1) and six (T2) months post-stroke. The GHQ-28, administered as a highly structured interview, was the primary outcome measurement of the RCT along with five secondary outcome measurements and the registration of demographic data. The data collection were conducted in the participants’ homes or wherever the participants were at the time of the assessment. The assessor read the questions to the respondent, and recorded the respondent’s answers in a web-based secure questionnaire by using a tablet.

### GHQ-28

To evaluate the effect of the psychosocial intervention on well-being, the GHQ-28 was chosen as the primary outcome based on results from a comparable trial and because it was evaluated as an appropriate tool to capture emotional stress [5]. The GHQ-28 requests participants to indicate how their health in general has been over the past few weeks, using behavioural items with a 4-point scale indicating the following frequencies of experience: “not at all”, “no more than usual”, “rather more than usual” and “much more than usual”. The scoring system applied in this study was the same as the original scoring system [6], the Likert scale 0, 1, 2, 3 [21]. The minimum score for the 28 version is 0, and the maximum is 84. Higher GHQ-28 scores indicate higher levels of distress. Goldberg suggests that participants with total scores of 23 or below should be classified as non-psychiatric, while participants with scores > 24 may be classified as psychiatric, but this score is not an absolute cut-off. It is recommended that each researcher derive a cut-off score based on the mean of their respective sample [22].

### Statistics

Exploratory factor analysis (EFA) was performed using SPSS Statistics for Windows, Version 24.0 [23]. Monte Carlo PCA was used for the parallel analysis [24]. The lavaan package version 0.5–23 [25] in R version 3.03 [26] was used to conduct the confirmatory factor analysis (CFA) and the analysis of metric invariance.

The minimum amount of data for factor analysis was satisfied [27, 28], with a final sample size of 322 (complete cases) for the exploratory factor analysis at time point T1 (providing a ratio of 11.5 cases per variable). The 285 complete cases with data from both T1 and T2 were used for the CFA (providing a ratio of 10.2 cases per variable).

The data were screened for outliers, skewness and missing values. The missing values were treated as missing at random (MAR). Using multiple imputation by chained equations (MICE) in SPSS, the single missing items where imputed at both time points [29, 30]. The MICE imputation model was constructed to include each of the 28 single items across time points both as predictors and to be imputed using the SPSS default imputation method of linear regression. Item constraints were limited according to the Likert-scoring method and imputation was specified to the closest integer. The multiple imputation produced five imputed data sets. Because we only use the T1 data for the EFA and exclude the cases completely missing at T2 for the CFA, missing values were minimal (< 1% for both time points). The result are therefore only presented from one (imputation 1) imputed dataset instead of pooled results of the five imputed datasets, which is an acceptable approach for very low proportions of missing data (< 3%) [31].

Initially, the factorability of the questionnaire was examined. Several criteria for the factorability of a correlation were used. The correlation matrix was examined for correlations above 0.3 [28]. The Kaiser-Meyer-Olkin (KMO) measure was used to test the sampling adequacy and was required to exceed 0.60 [32]. The result of Bartlett’s test of sphericity [33] was considered statistically significant if the *p*-value was < 0.05. Cronbach’s alpha was used to estimate the reliability of the instruments based on a required internal consistency > 0.7 [27, 34].

The factor structure was explored by EFA prior to evaluating construct validity by CFA. The EFA was conducted using the unweighted least squares method with direct oblimin rotation with Kaiser normalization to account for correlations between the items [28].

The number of factors to be retained was guided by three decision rules: Kaiser’s criterion (eigenvalue > 1), inspection of the scree plots, and Horn’s parallel analysis [24]. Parallel analysis has been shown to provide more consistent results when estimating the number of components than the more traditional methods based on eigenvalue > 1 and scree plots alone [27]. Only factors with eigenvalues that exceeded the corresponding values from the random dataset in the parallel analysis were retained. As recommended, only factors loading greater than 0.30 were displayed, making the output easier to interpret [27].

CFA, using maximum likelihood estimation was conducted to evaluate the model fit to the original construct of the GHQ-28 as proposed by Goldberg et.al [6], by examining if indicators of selected constructs loaded onto separate factors in the expected manner [35]. The analysis was performed by group using data from both the baseline and six-month datasets.

Several goodness-of-fit indicators were considered in the analyses. Comparative fit index (CFI) and Tucker-Lewis index (TLI) values less than .95 indicated lack of fit, and values above .95 indicated good fit [28, 36]. A root mean squared error of approximation (RMSEA) of .06 or lower is suggested to indicate a good fit [36].

We assessed measurement invariance by investigating three levels of invariance, as recommended in previous studies [37, 38]. The most basic level of measurement invariance is configural invariance, which assumes that the items load on the same latent factors across groups, but factor loadings can vary. The second level, metric invariance, requires that all factor loadings are the same across groups. Scalar invariance is the strongest form of invariance; it implies metric invariance and in addition tests if the intercepts are the same across the two time points. A change in CFI of less than 0.01 was considered evidence of invariance. This cut-off is based on the cut-off value used in a comparable study [17] and recommendations [36].

## Results

### Sample characteristics

**Fig. 1:**
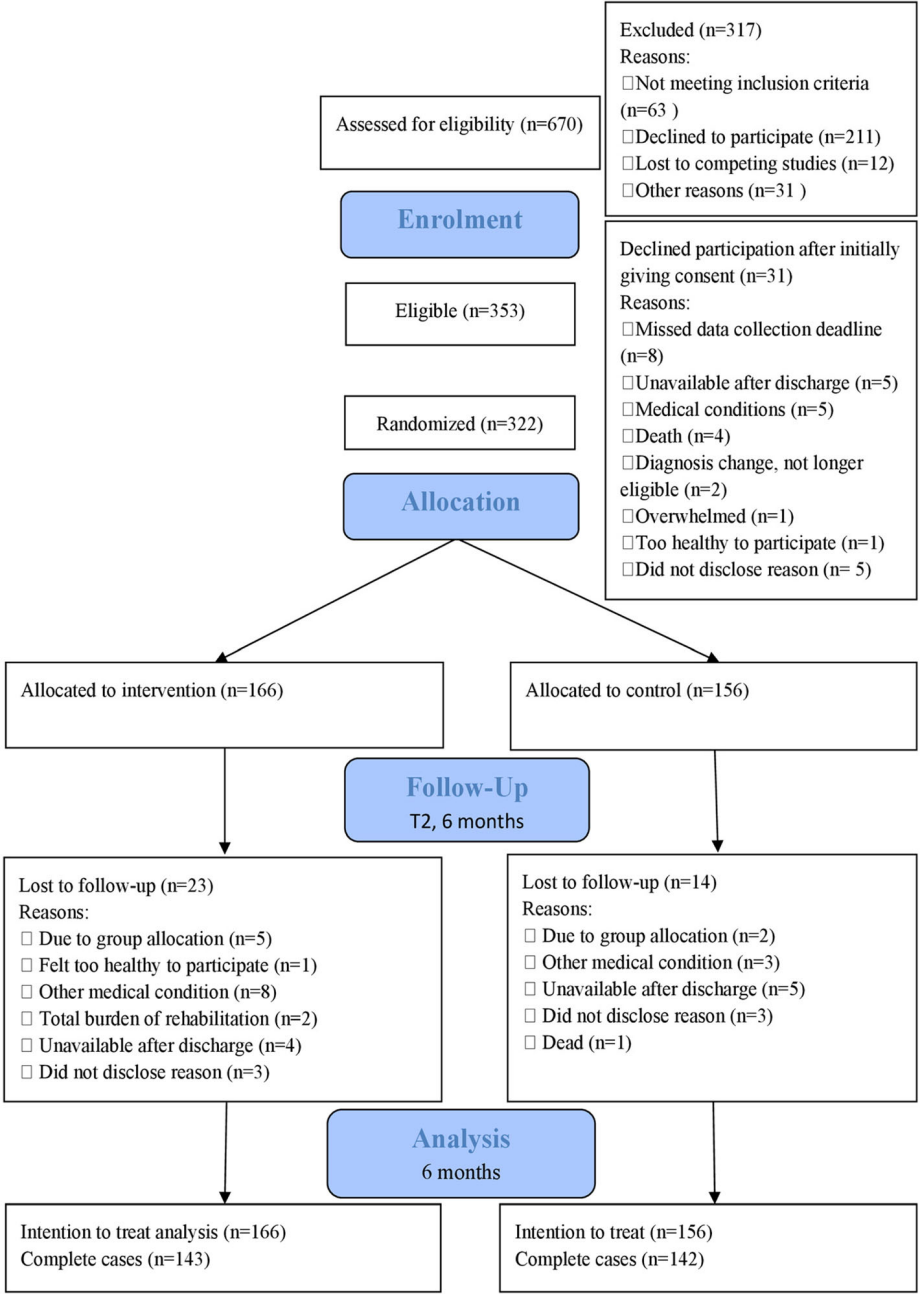
CONSORT diagram of the flow of patients through the trial

The flow of participants is shown in Fig. 1 and the characteristics of the 322 randomized are shown in Table 1. The age ranged from 20 to 90 years, with a mean age of 66.2 years (SD 12.6). There were more males (59%) than females (41%) participating in the study. According to the measurement of neurological deficits, National Institutes of Health Stroke Scale (NIHSS), among the participants for whom we have information, 70% had no or minor symptoms (scoring between 0 and 5 on the NIHSS). In addition, based on the national register for stroke patients admitted to hospitals in Norway, our participants are on average 8 years younger than the national stroke population. We have 5% more men than expected based on the stroke population in Norway and fewer patients with higher stroke severity [39].

**Table 1:** Characteristics at baseline (T1) and data from the Norwegian stroke population

	Mean (SD)/ Total (%)	The Norwegian stroke register ^a^
Age		
Mean (SD)	66.2 (12.6)	74.4
Median	67	76
Range	20–90	19–104
*Missing*	0	-
Gender		
Female	132 (41%)	3895 (46%)
Male	190 (59%)	4514(54%)
*Missing*	0	-
National Institutes of Health Stroke Scale (NIHSS) ^b^		
0–5	170 (70%)	4119 (65%)
6–10	45 (19%)	1009 (16%)
11–15	17 (7%)	505 (8%)
16 +	8 (4%)	675 (11%)
*Missing*	82 (25%)	2230 (26%)
GHQ-28 sum score		
GHQ-28 (T1) Min 6, Max 72	27 (11.4)	-
*Complete cases missing*	0	-
GHQ-28 (T2 (*n* = 285)) Min 5, Max 60	20 (10.2)	-
*Complete cases missing*	37 (11%)	-

^a^ Data from the Norwegian stroke population admitted to hospitals in 2015 registered in a Norwegian stroke register [39]. ^b^ Of the 240 patients for whom we had baseline data and the 6308 for whom data were registered in the Norwegian stroke register

At 1 month post-stroke (T1), the sum scores on the GHQ-28 ranged from 6 to 72, with a mean sum score of 27 (SD 11.4). At 6 months post-stroke (T2), the sum scores ranged from 5 to 60, with a mean sum score of 20 (SD 10.2).

There were few missing values in the dataset, representing only 0.29% of the 11 total values for the single items at T1, and there were no complete missing cases. The total percentage of missing values at T2 was 11.6% measured in single items; however, after excluding the 37 complete missing cases, the percentage of missing values was only 0.09%.

The 37 participants that were lost to follow up at T2, did not have higher mean score on GHQ-28 compared to the 285 with data from both time points, but the mean age were higher (5 years) and they reported more severe symptoms, more depression and more experiences of fatigue. However, only data from participants that were assessed at both T1 and T2 was used for the CFA. Since we are comparing the same set of patients at T1 and T2, the results are comparable regardless of considering potential higher severity and consequences of stroke for the participants missing at T2.

### Exploratory factor analysis (EFA)

#### No forced factors

**Fig. 2:**
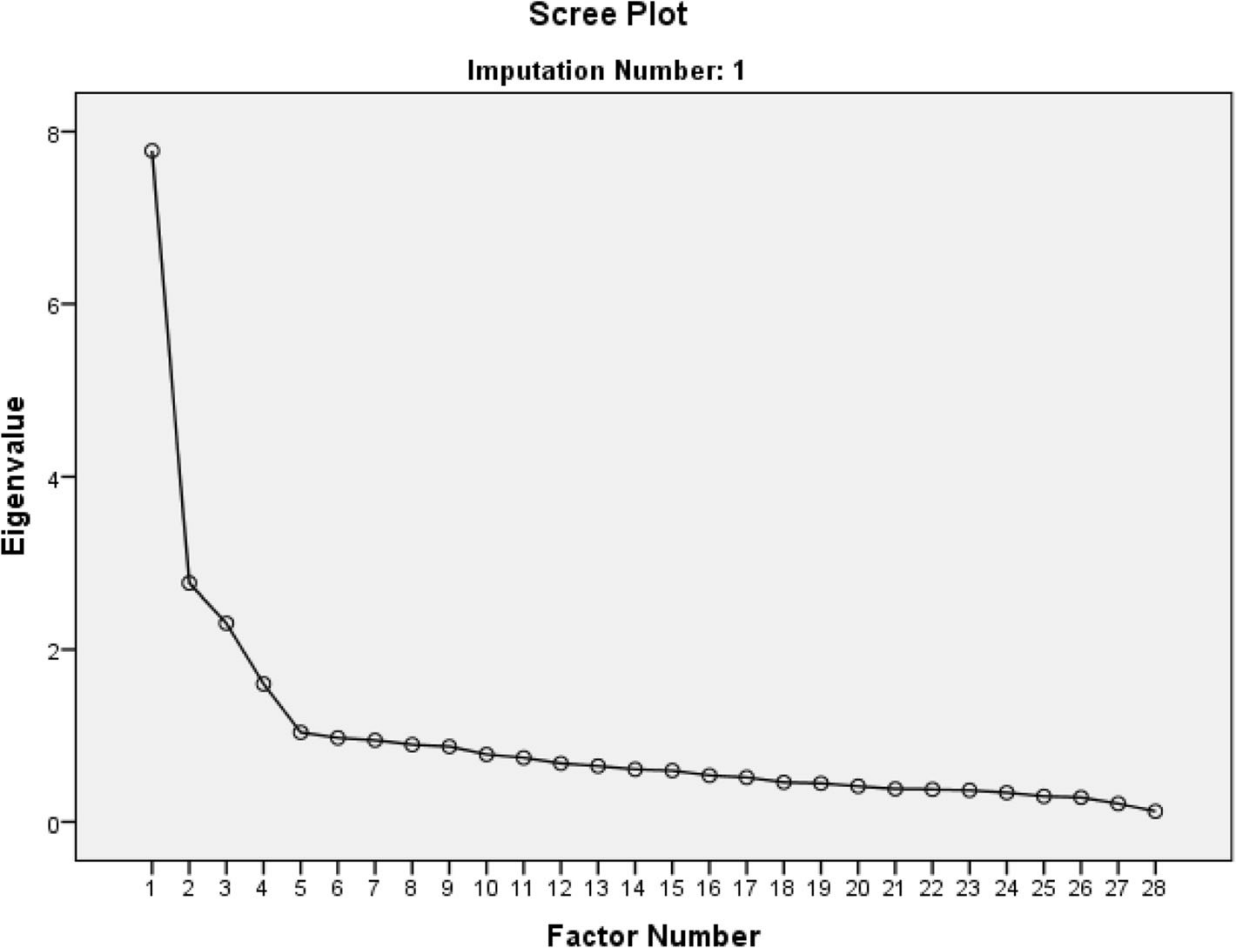
Screeplot from the EFA with no forced factors

The exploratory analysis of the imputed dataset, with no forced factors, resulted in five factors exceeding an eigenvalue of one, and the scree plot showed a change in the curve after five factors (Fig. 2).

Horn’s parallel analysis (Table 2) showed that only four components exceeded the corresponding criterion value for a randomly generated data matrix of the same size (28 variables × 322 respondents).

**Table 2:** Horn’s parallel analysis of the five factors exceeding an eigenvalue of 1

Component number	Actual eigenvalue from the EFA at T1	Criterion value from the parallel analysis	Decision
1	7.795	1.589	Accept
2	2.772	1.497	Accept
3	2.302	1.433	Accept
4	1.596	1.381	Accept
5	1.038	1.331	Reject

Based on these analyses, four factors were retained for further EFA.

#### Four forced factors

Inspection of the correlation matrix revealed that all 28 items correlated > .3 with at least one other factor. There were significant positive correlations among the four latent factors (Table 3) supporting the use of oblique (oblimin) rotation [28] and indicating that respondents who showed high level in one dimension were more likely to show high level in the others as well.

**Table 3:** Factor correlation matrix ^a^

Factor	1	2	3	4
1	1000	–0,380	–0,459	0,279
2	–0,380	1000	0,259	0,017
3	–0,459	0,254	1000	-0,157
4	0,279	0,017	-0,157	1000

Extraction Method: Unweighted Least Squares. Rotation Method: Oblimin with Kaiser Normalization, Imputation 1

^a^If correlations between factors are > 0.3, oblique rotation is the recommended approach because it produces a clearer result than orthogonal

The KMO measure was 0.883, and Bartlett’s test of sphericity reached statistical significance (*p* < 0.001) supporting the suitability for factor analysis.

The rotated solution revealed a structure with a number of strong loadings > .45 [28]. Only five of the included variables loaded less than .45 (.34–.44). All the variables loaded substantially on one component.

The four-component solution explained a total of 51.6% of the variance at 1 month, with Factor 1 contributing to 27.8%, factor 2 contributing to 9.9%, factor 3 contributing to 8.2% and factor 4 contributing to 5.7%. Details from the analysis are listed in Table 4.

**Table 4:** Exploratory factor analysis (EFA) with four forced factors (*n* = 322, Imputation 1)

	Factor 1 Explaining 27.8% of the variance Cronbach’s α: 0.844		Factor 2 Explaining 9.9% of the variance Cronbach’s α: 0.881		Factor 3 Explaining 8.2% of the variance Cronbach’s α: 0.838		Factor 4 Explaining 5.7% of the variance Cronbach’s α: 0.719		
	Pattern	Structure	Pattern	Structure	Pattern	Structure	Pattern	Structure	^a^
(A) Somatic symptoms									
1. Been feeling perfectly well and in good health?					−0.742	−0.694			0.457
2. Been feeling in need of a good tonic?					−0.364	−0.430			0.270
3. Been feeling run down and out of sorts?					−0.514	−0.569			0.378
4. Been feeling that you are ill?					−0.491	−0.568			0.432
5. Been getting any pains in your head?							0.718	0.754	0.535
6. Been getting a feeling of tightness or pressure in your head?							0.637	0.677	0.518
7. Been having hot or cold spells?							0.448	0.508	0.320
(B) Anxiety and insomnia									
1. Been losing much sleep over worry?	0.572	0.610							0.414
2. Been having difficulty in staying asleep once you fall asleep?	0.344	0.433							0.321
3. Been feeling constantly under strain?	0.585	0.585							0.372
4. Been getting edgy or bad tempered?	0.485	0.508							0.327
5. Been getting scared or panicky for no reason?	0.635	0.612							0.444
6. Been feeling everything is getting on top of you?	0.621	0.659							0.442
7. Been feeling nervous and strung-out all the time?	0.710	0.713							0.482
(C) Social dysfunction									
1. Been managing to keep yourself busy and occupied?					−0.521	−0.553			0. 381
2. Been taking longer over the things you do?					−0.670	−0.644			0.427
3. Been feeling on the whole that you were doing things well?					−0.692	−0.689			0.480
4. Been satisfied with the way you have carried out your tasks?					−0.688	−0.716			0.499
5. Been feeling that you are playing a useful part in things?					−0.646	−0.643			0.439
6. Been feeling capable of making decisions about things?					−0.349	−0.403			0.220
7. Been able to enjoy your normal day-to-day activities?	0.392	0.442							0.327
(D) Severe depression									
1. Been thinking of yourself as a worthless person?	0.515	0.589							0.469
2. Been feeling that life is entirely hopeless?	0.580	0.670							0.584
3. Been feeling that life is not worth living?			−0.591	−0.699					0.560
4. Been thinking of the possibility that you may do away with yourself?			−0.974	−0.957					0.827
5. Been feeling at times that you could not do anything because your nerves were too bad?	0.493	0.596							0.480
6. Been finding yourself wishing you were dead and away from it all?			−0.827	−0.856					0.730
7. Been finding that the idea of taking your own life keeps coming into your mind?			−0.869	−0.835					0.726

^a^Communalities indicate the amount of variance in each variable that is accounted for

The Norwegian version of the GHQ-28 was internally consistent, as indicated by Cronbach alpha values of 0.844, 0.881, 0.838 and 0.719 for the four subscales.

Inspection of the pattern matrix shows that all the anxiety and insomnia questions cluster together, accompanied by one question from the social dysfunction subscale and three from the severe depression subscale. Only four questions remain in the severe depression factor. The questions regarding somatic symptoms cluster together with six of the questions from the social dysfunction subscale. The three questions concerning headaches or having hot or cold spells form their own category.

Overall, these results support a four-factor solution as proposed by Goldberg and Hillier [6]. However, the content of the factors does not fully support the original scale structure. This finding makes it difficult to confirm the original factor composition by examining the results of the EFA alone. Therefore, the next step taken was to test, by means of CFA, the fit of the original structure in our stroke sample.

### Confirmatory factor analysis (CFA)

We fit the model using the full information maximum likelihood (FIML). The comparative fit indices (CFI and TLI) did not reach the level of 0.95, which would indicate a good fit [28, 36]. The root mean squared error of approximation (RMSEA), which assesses the extent to which a model fits reasonably well in a population [35], exceeded the recommended fit index of 0.06 by 0.02. By this, we could not confirm construct validity. The fit indices are listed in Table 5.

**Table 5:** Fit indices and estimates of the latent variable for the T1 and T2 datasets (imputation 1) (n = 285)

Items	T1	T2
χ 2 (df)	p < 0.001 (378)	p < 0.001 (378)
CFI^b^	0.784	0.774
TLI	0.762	0.752
RMSEA	0.084	0.088
Latent variables^a^		
(A) Somatic symptoms		
Item 1	0.518	0.399
Item 2	0.452	0.457
Item 3	0.627	0.634
Item 4	0.702	0.535
Item 5	0.521	0.337
Item 6	0.501	0.475
Item 7	0.379	0.300
(B) Anxiety and insomnia		
Item 1	0.623	0.528
Item 2	0.390	0.416
Item 3	0.480	0.383
Item 4	0.442	0.449
Item 5	0.522	0.414
Item 6	0.570	0.499
Item 7	0.589	0.506
(C) Social dysfunction		
Item 1	0.449	0.406
Item 2	0.451	0.415
Item 3	0.413	0.365
Item 4	0.562	0.477
Item 5	0.453	0.414
Item 6	0.213	0.172
Item 7	0.333	0.269
(D) Severe depression		
Item 1	0.385	0.414
Item 2	0.436	0.480
Item 3	0.465	0.442
Item 4	0.410	0.347
Item 5	0.335	0.343
Item 6	0.413	0.456
Item 7	0.354	0.365

^a^All the estimates had a p-value < 0.001

^b^*CFI* comparative fit index, *TLI* Tucker–Lewis index, *RMSEA* root mean square error of approximation

### Measurements of invariance

The results from the testing of measurement invariance showed that the GHQ-28 questionnaire has comparable measurement properties at T1 and T2. The fit of the least restrictive configural invariance model was compared with the results from the more restrictive metric and scalar invariance models (Table 6). Neither the metric nor scalar invariance model produced a change in the CFI of ≥0.01, which confirmed the metric and scalar measurement invariance within groups for the two time points.

**Table 6:** Overall fit indices from the measurement invariance tests

Measurement invariance model^a^	χ2(*df*)	CFI	TLI	RMSEA
Configural	2143.235 (688) p < 0.001	0.779	0.757	0.086
Metric	2176.377 (716) p < 0.001	0.778	0.766	0.085
Scalar	2262.083 (740) p < 0.001	0.769	0.764	0.085

^a^*CFI* comparable fit index, *TLI* Tucker-Lewis index, *RMSEA* root mean square error of approximation

## Discussion

The aim of the study was to explore the psychometric properties of the GHQ-28 when applied in a Norwegian stroke population by evaluating the internal consistency, exploring the factor structure, construct validity and measurement invariance.

Overall, the results from the EFA support a four-factor solution, but some of the items load on different factors from those in the original version proposed by Goldberg and Hillier [6]. The often-suggested threshold for the indices of goodness of fit in a CFA was not achieved, which indicates that caution is required when interpreting subfactor scores in a stroke sample. Measurement invariance was established for the same groups over two time points, which has, to the best of our knowledge, not previously been evaluated for GHQ-28 in a stroke population. This confirms that the same construct is being measured at both time points.

The EFA shows that the first factor in our sample addresses issues concerning anxiety and insomnia, in addition to one item from the social dysfunction subscale regarding enjoyment of daily activities and three items regarding nervousness and feelings of hopelessness originally categorized in the severe depression subcategory. category"?>. This finding reflects the correlation between anxiety and depressive symptoms, which are known to be associated with one another in a stroke population [40, 41].

The second factor consists of the four most severe questions from the severe depression category about lack of joy in life and suicidality. The severity of the questions distinguishes them from the other questions regarding less severe depressive thoughts that correlate with anxiety and insomnia. Because the questions that address depressive thoughts are split between two factors in this study, examining the scoring in the original severe depression category alone is not sufficient when evaluating depression in a stroke population.

The third category contains four items from the original somatic symptoms factor and six items from the social dysfunction factor. Not feeling “perfectly well and in good health” in addition to feelings of being “run down and out of sorts”, “in need of a good tonic” or having “feelings of being ill” are, not unexpectedly, associated with social dysfunction. Altogether, these seven subjective evaluation questions address factors of social interrelation, emotional reactions, and judgements formed about life satisfaction and fulfilment, which can be interpreted as aspects of social function and psychosocial well-being.

The original population in which the measurement was developed did not suffer from any specific somatic illnesses. It has previously been claimed that certain responses on the GHQ-28 can be produced by physical or psychiatric disease [8, 42, 43]. In our study, an example of this situation is particularly apparent when we investigate the fourth factor from the EFA. This factor is formed by the items addressing somatic symptoms such as headache or having hot or cold spells. Pain and headache is a complication that can occur after stroke [44] and may also be a known side effect of medications used as secondary prevention after stroke [45] and is therefore not necessarily related to psychological distress. Even if an association with psychological challenges can be argued, forming a separate category, this does not necessarily make the items irrelevant to the evaluation of psychosocial well-being using the GHQ-28 total score since pain is known to be associated with health-related quality of life [46].

There are challenges applying a rating scale across countries and languages and to different populations. The stability of the factor structure has been examined in a study comparing the results from several different countries [10]. The researchers highlight some factors that might explain the differences as variances in the expression of distress, effect of translation and degree of industrial development. In our sample, most of the participants were born in Norway to Norwegian parents (92%). Even if the sample in this study is homogeneous, the original factor structure was developed in a London cultural setting. Subtle changes in understanding due to linguistic nuances or cultural differences in beliefs about health, expectations for the rehabilitation process or health care system may influence how the questionnaire was scored.

A strength in this study is that there were few missing items. Another strength is the application of both exploratory and confirmatory factor analyses.

One limitation is not having a sufficient sample to split the material for the EFA and CFA. Another limitation is that the patients with the most severe strokes or aphasia were difficult to enrol due to early inclusion and requirements for informed consent. Nevertheless, the study sample is representative of a large amount of the stroke population in Norway, since mild and moderate strokes are far more common than severe strokes [39].

## Conclusions

The Norwegian version of the GHQ-28 confirms a four-factor solution, but with some differences in the factor structure compared to that of the original version. The CFA did not reach the strict cut-off for goodness of fit recommended in the literature. Measurement invariance across time points was confirmed, indicating that the same construct of the GHQ-28 is measured across time. However, the change in mean score on the GHQ-28 and the factor composition are assumed to be affected by characteristics in the stroke population. The results, when applying GHQ-28 in a stroke population, and sub-factor analysis based on the original factor structure should be interpreted with caution.

## Abbreviations

CFA: Confirmatory factor analysis
CFI: Comparative fit index
EFA: Exploratory factor analysis
GHQ - 28: General Health Questionnaire - 28
KMO: Kaiser-Meyer-Olkin
NIHSS: National Institutes of Health Stroke Scale
RMSEA: Root mean square error of approximation
TLI: Tucker–Lewis index
